# Supplementary material for: The relationship between antihypertensive medications and mood disorders: analysis of linked healthcare data for 1.8 million patients
Source: Psychol Med. 2020 Jan 24;51(7):1183–91. doi: 10.1017/S0033291719004094 (PMC8188528; doi:10.1017/S0033291719004094)
Supplement: Supplementary file 1 [file S0033291719004094sup001.docx]

**Online tables and figures**

Table S1: BNF Codes (Chapter.Section.Paragraph.Subparagraph), headings and example drugs.

| BNF Codes | Headings | Example drugs |
| --- | --- | --- |
| 2 | **Cardiovascular system** |  |
| 2.1 | Positive Inotropic | Digoxin, Enoximone |
| 2.2 | Diuretics | Bumetanide, furosemide |
| 2.2.1 | Thiazides and related diuretics | Bendroflumethiazide,  Cyclopenthiazide |
| 2.3 | Anti-arrhythmic drugs | Adenosine |
| 2.4 | Beta-adrenoceptor blocking drugs | Atenolol, Propranolol, Pindolol |
| 2.5.5 | Drugs Affecting the renin-angiotensin system |  |
| 2.5.5.1 | Angiotensin-converting enzyme inhibitors | Captopril, Enalapril maleate, Lisinopril |
| 2.5.5.2 | Angiotensin-II receptor antagonists | Candesartan Cilexetil, Losartan potassium |
| 2.5.5.3 | Renin Inhibitors | Aliskiren |
| 2.6 | Nitrates, calcium-channel blockers, and other antianginal drugs |  |
| 2.6.1 | Nitrates | Isosorbide Mononitrate |
| 2.6.2 | Calcium-channel blockers | Amlodipine, Felodipine, Nifedipine |
| 2.6.3 | Other antianginal drugs | Ivabradine |
| 2.6.4 | Peripheral vasodilators and related drugs | Cilostazol, Moxisylyte |
| 2.9 | Antiplatelet drugs | Asprin, Clopidogrel |
| 2.12 | Lipid-regulating drugs | Pravastatin sodium |
| 4 | **Central nervous system** |  |
| 4.1 | Hypnotics and anxiolytics | Benzodiazepines |
| 4.2 | Drugs used in psychoses and related disorders | Clozapine, Benperidol |
| 4.3 | Antidepressant drugs | Selective serotonin re-uptake inhibitors, Tricyclics |
| 6 | **Endocrine System** |  |
| 6.1.2 | Antidiabetic drugs | Sulfonylureas, Biguanides |

Table S2: Descriptive statistics for unmatched cohort 1 members

|  |  | Thiazide diuretics | Beta Blockers | Angiotensin Antagonists | Calcium Channel Blockers | Polytherapy | Other Antiypertensives |
| --- | --- | --- | --- | --- | --- | --- | --- |
| N of observations |  | 13,001 | 15,721 | 28,536 | 15,996 | 130,443 | 16,683 |
|  |  |  |  |  |  |  |  |
| **Gender** |  | % | % | % | % | % | % |
| Male |  | 32.3 | 50.9 | 51.8 | 49.3 | 49.1 | 36.0 |
| Female |  | 67.7 | 49.1 | 48.2 | 50.7 | 50.9 | 64.1 |
| **Survival Events** |  |  |  |  |  |  |  |
| Treatment for depression |  | 26.6 | 25.6 | 26.6 | 25.0 | 25.6 | 27.2 |
| Treatment for Bipolar disorder Yes |  | 7.3 | 8.0 | 7.2 | 7.3 | 6.6 | 9.7 |
| Died |  | 28.4 | 34.9 | 35.3 | 35.6 | 32.7 | 52.3 |
| Changed therapy |  | 47.7 | 36.2 | 39.4 | 39.1 | 0.0 | 0.0 |
| **History of hospital treatment** |  |  |  |  |  |  |  |
| Cardiovascular disease |  | 8.0 | 41.4 | 27.0 | 24.0 | 32.9 | 30.5 |
| Head Injury |  | 1.4 | 1.5 | 1.6 | 1.8 | 1.4 | 1.7 |
| Substance abuse |  | 0.8 | 1.3 | 1.1 | 1.4 | 1.1 | 1.1 |
| Self-harm |  | 0.0 | 0.1 | 0.1 | 0.1 | 0.0 | 0.1 |
| **History of pharmaceutical treatment** |  |  |  |  |  |  |  |
| Other Cardiovascular meds |  | 53.8 | 77.8 | 77.0 | 68.3 | 79.8 | 69.5 |
| Diabetic Drugs |  | 4.1 | 5.8 | 18.1 | 5.4 | 14.3 | 9.7 |
| **Age** |  |  |  |  |  |  |  |
| Mean |  | 76.9 | 76.4 | 76.1 | 76.8 | 76.2 | 79.6 |
| SD |  | 7.2 | 7.8 | 7.7 | 7.7 | 7.0 | 7.8 |
| **SIMD** |  |  |  |  |  |  |  |
| Mean |  | 5.4 | 5.3 | 5.2 | 5.2 | 5.2 | 5.2 |
| SD |  | 2.7 | 2.7 | 2.7 | 2.7 | 2.7 | 2.7 |
|  |  |  |  |  |  |  |  |

Table S3: Descriptive statistics for unmatched cohort 2 members

|  | Thiazide diuretics | Beta Blockers | Angiotensin Antagonists | Calcium Channel Blockers | Polytherapy | Other Antiypertensives |
| --- | --- | --- | --- | --- | --- | --- |
|  |  |  |  |  |  |  |
| N of observations | 10,125 | 22,292 | 27,999 | 15,916 | 98,283 | 23,717 |
|  | | | | | | |
| *Categorical variables* | | | | | | |
|  | % | % | % | % | % | % |
| **Gender** |  |  |  |  |  |  |
| Male | 20.4 | 38.5 | 41.8 | 36.5 | 40.9 | 29.8 |
| Female | 79.6 | 61.5 | 58.2 | 63.5 | 59.1 | 70.2 |
| **Survival Events** |  |  |  |  |  |  |
| Treatment for MDD | 74.4 | 73.9 | 73.1 | 71.7 | 72.3 | 69.6 |
| Treatment for Bipolar disorder | 14.2 | 16.0 | 14.2 | 16.2 | 11.6 | 19.1 |
| Died | 28.8 | 28.5 | 34.1 | 37.2 | 30.3 | 52.5 |
| Changed therapy | 45.1 | 28.4 | 37.6 | 35.1 | 0.0 | 0.0 |
| **History of hospitalization** |  |  |  |  |  |  |
| Cardiovascular | 12.4 | 34.0 | 31.4 | 33.7 | 41.1 | 34.2 |
| Substance Abuse | 9.6 | 18.2 | 15.7 | 16.9 | 16.8 | 17.9 |
| Head injury | 2.3 | 4.4 | 3.8 | 4.2 | 3.3 | 3.7 |
| Self Harm | 2.3 | 5.0 | 3.0 | 3.3 | 2.7 | 3.1 |
| Schizophrenia | 1.8 | 2.4 | 1.9 | 2.2 | 1.4 | 2.7 |
| Major Depressive Disorder | 6.2 | 9.6 | 8.0 | 8.4 | 6.3 | 9.9 |
| Bipolar Disorder | 1.0 | 1.6 | 1.3 | 1.5 | 0.8 | 1.4 |
| Other mood disorder | 1.0 | 1.7 | 1.4 | 1.7 | 1.1 | 1.6 |
| Personality disorder | 0.6 | 1.4 | 0.8 | 1.1 | 0.7 | 1.1 |
| **History of prescriptions** |  |  |  |  |  |  |
| Cardiovascular | 53.1 | 62.2 | 75.5 | 70.9 | 81.2 | 66.3 |
| Diabetic drugs | 5.1 | 6.3 | 20.1 | 6.7 | 16.4 | 10.1 |
| hypnotics | 41.4 | 44.3 | 38.5 | 42.6 | 39.2 | 46.0 |
| Antidepressants | 68.1 | 68.2 | 66.8 | 65.2 | 64.4 | 64.7 |
| Psychoses and related drugs | 7.2 | 9.3 | 7.1 | 8.4 | 4.8 | 11.4 |
|  |  |  |  |  |  |  |
| *Continuous Variables* | | | | | | |
| **Age** |  |  |  |  |  |  |
| Mean | 71.5 | 66.7 | 69.5 | 71.7 | 69.9 | 74.2 |
| SD | 10.3 | 13.1 | 11.3 | 10.6 | 10.0 | 11.5 |
| **SIMD** |  |  |  |  |  |  |
| Mean | 4.9 | 4.7 | 4.7 | 4.6 | 4.7 | 4.8 |
| SD | 2.8 | 2.8 | 2.7 | 2.7 | 2.7 | 2.7 |
|  |  |  |  |  |  |  |

“Other Antihypertensives” were defined on the basis of treatment with a combination of thiazide diuretics, diuretics, BBs, AA and/or CCBs, but not treatment with at least two of these groups within in the last 3 months of the eligible treatment window.

Table S4: Frequency and percentage of the various combination of prescriptions in the “Other Antihypertensives” class

|  |  |  |  |  |  | Cohort 1 | |  | Cohort 2 | |
| --- | --- | --- | --- | --- | --- | --- | --- | --- | --- | --- |
| Other Diuretics | Thiazide diuretics | Beta Blockers | Angiotensin Antagonists | Calcium Channel Blockers |  | N. | % |  | N. | % |
| 0 | 0 | 0 | 1 | 1 |  | 4,624 | 11.62 |  | 1,945 | 5.68 |
| 0 | 0 | 1 | 0 | 1 |  | 1,482 | 3.72 |  | 1,293 | 3.77 |
| 0 | 0 | 1 | 1 | 0 |  | 2,588 | 6.50 |  | 1,797 | 5.25 |
| 0 | 0 | 1 | 1 | 1 |  | 315 | 0.79 |  | 187 | 0.55 |
| 0 | 1 | 0 | 0 | 1 |  | 1,915 | 4.81 |  | 679 | 1.98 |
| 0 | 1 | 0 | 1 | 0 |  | 2,493 | 6.26 |  | 846 | 2.47 |
| 0 | 1 | 0 | 1 | 1 |  | 674 | 1.69 |  | 262 | 0.76 |
| 0 | 1 | 1 | 0 | 0 |  | 635 | 1.60 |  | 422 | 1.23 |
| 0 | 1 | 1 | 0 | 1 |  | 102 | 0.26 |  | 61 | 0.18 |
| 0 | 1 | 1 | 1 | 0 |  | 258 | 0.65 |  | 92 | 0.27 |
| 0 | 1 | 1 | 1 | 1 |  | 94 | 0.24 |  | 58 | 0.17 |
| 1 | 0 | 0 | 0 | 0 |  | 15,652 | 39.33 |  | 18,870 | 55.09 |
| 1 | 0 | 0 | 0 | 1 |  | 1,030 | 2.59 |  | 985 | 2.88 |
| 1 | 0 | 0 | 1 | 0 |  | 2,583 | 6.49 |  | 1,986 | 5.80 |
| 1 | 0 | 0 | 1 | 1 |  | 391 | 0.98 |  | 220 | 0.64 |
| 1 | 0 | 1 | 0 | 0 |  | 2,792 | 7.01 |  | 2,953 | 8.62 |
| 1 | 0 | 1 | 0 | 1 |  | 155 | 0.39 |  | 119 | 0.35 |
| 1 | 0 | 1 | 1 | 0 |  | 427 | 1.07 |  | 250 | 0.73 |
| 1 | 0 | 1 | 1 | 1 |  | 67 | 0.17 |  | 41 | 0.12 |
| 1 | 1 | 0 | 0 | 0 |  | 833 | 2.09 |  | 812 | 2.37 |
| 1 | 1 | 0 | 0 | 1 |  | 139 | 0.35 |  | 77 | 0.22 |
| 1 | 1 | 0 | 1 | 0 |  | 270 | 0.68 |  | 139 | 0.41 |
| 1 | 1 | 0 | 1 | 1 |  | 89 | 0.22 |  | 30 | 0.09 |
| 1 | 1 | 1 | 0 | 0 |  | 110 | 0.28 |  | 92 | 0.27 |
| 1 | 1 | 1 | 0 | 1 |  | 21 | 0.05 |  | 12 | 0.04 |
| 1 | 1 | 1 | 1 | 0 |  | 39 | 0.10 |  | 17 | 0.05 |
| 1 | 1 | 1 | 1 | 1 |  | 23 | 0.06 |  | 11 | 0.03 |
|  |  |  |  |  |  |  |  |  |  |  |

NB 0 and 1 indicated absence or presence or of a particular antihypertensive treatment

Table S5: Sociodemographic and medical event and comorbidity variables that are available for both cohorts

|  | Controls |  | Thiazide diuretics  (Thiazide) | |  | Beta Blockers (BB) | |  | Angiotensin Antagonists  (AA) | |  | Calcium Channel Blockers  (CCBs) | |  | Polytherapy  (Poly) | |  | Other Antihypertensives | |
| --- | --- | --- | --- | --- | --- | --- | --- | --- | --- | --- | --- | --- | --- | --- | --- | --- | --- | --- | --- |
|  | Cohort 2 |  | Cohort 1 | Cohort 2 |  | Cohort 1 | Cohort 2 |  | Cohort 1 | Cohort 2 |  | Cohort 1 | Cohort 2 |  | Cohort 1 | Cohort 2 |  | Cohort 1 | Cohort 2 |
|  |  |  |  |  |  |  |  |  |  |  |  |  |  |  |  |  |  |  |  |
| Total sample size (n) | 502,937 |  | 25,555 | 8,477 |  | 73,996 | 90,919 |  | 130,110 | 48,408 |  | 57,986 | 24,634 |  | 211,282 | 65,584 |  | 39,801 | 34,256 |
| **Gender** |  |  |  |  |  |  |  |  |  |  |  |  |  |  |  |  |  |  |  |
| Male (%) | 52.8 |  | 29.8 | 21.4 |  | 44.2 | 44.7 |  | 60.3 | 46.2 |  | 53.7 | 39.2 |  | 56.4 | 48.4 |  | 40.9 | 33.7 |
| Female (%) | 15.6 |  | 70.2 | 78.6 |  | 55.8 | 15.0 |  | 39.7 | 53.8 |  | 46.3 | 60.8 |  | 43.6 | 51.6 |  | 59.1 | 66.3 |
|  |  |  |  |  |  |  |  |  |  |  |  |  |  |  |  |  |  |  |  |
| **Survival events** |  |  |  |  |  |  |  |  |  |  |  |  |  |  |  |  |  |  |  |
| Treatment for depression (%) | 39.1 |  | 24.1 | 72.3 |  | 25.9 | 79.2 |  | 19.6 | 67.8 |  | 16.8 | 65.2 |  | 22.1 | 63.6 |  | 23.4 | 65.1 |
| Treatment for Bipolar disorder (%) | 8.0 |  | 2.7 | 10.6 |  | 1.7 | 11.6 |  | 1.4 | 8.3 |  | 2.0 | 9.9 |  | 2.1 | 7.3 |  | 4.4 | 15.5 |
| Died (%) | 9.6 |  | 8.7 | 12.5 |  | 5.6 | 4.6 |  | 5.1 | 7.2 |  | 7.1 | 9.8 |  | 8.9 | 9.4 |  | 22.1 | 9.8 |
| Changed Therapy (%) | na |  | 45.3 | 39.2 |  | 19.9 | 10.0 |  | 33.6 | 33.6 |  | 32.0 | 28.7 |  | NA | NA |  | NA | NA |
| **History of hospital treatment measure** |  |  |  |  |  |  |  |  |  |  |  |  |  |  |  |  |  |  |  |
| Cardiovascular disease (%) | 3.7 |  | 4.5 | 9.5 |  | 13.9 | 8.0 |  | 7.3 | 13.2 |  | 7.5 | 13.7 |  | 21.1 | 34.4 |  | 15.6 | 18.8 |
| Substance Abuse (%) | 16.8 |  | 0.9 | 12.6 |  | 1.3 | 18.0 |  | 1.3 | 18.8 |  | 1.4 | 19.1 |  | 1.3 | 28.2 |  | 1.5 | 27.0 |
| Head Injury (%) | 5.8 |  | 1.6 | 3.6 |  | 3.0 | 6.1 |  | 3.1 | 5.2 |  | 2.4 | 4.7 |  | 2.3 | 5.2 |  | 2.5 | 5.5 |
| Self-Harm (%) | 7.5 |  | 0.2 | 4.6 |  | 0.4 | 11.2 |  | 0.3 | 6.2 |  | 0.3 | 6.1 |  | 0.2 | 5.6 |  | 0.3 | 8.0 |
| **History of Prescriptions** |  |  |  |  |  |  |  |  |  |  |  |  |  |  |  |  |  |  |  |
| Other Cardiovascular drugs (%) | 14.3 |  | 39.2 | 40.8 |  | 39.7 | 21.2 |  | 45.8 | 51.1 |  | 44.3 | 49.6 |  | 60.6 | 64.3 |  | 48.3 | 45.5 |
| Diabetic drugs (%) | 2.2 |  | 3.3 | 4.6 |  | 3.2 | 2.6 |  | 15.4 | 17.8 |  | 3.9 | 4.9 |  | 11.1 | 9.8 |  | 7.3 | 7.6 |
| **Age** |  |  |  |  |  |  |  |  |  |  |  |  |  |  |  |  |  |  |  |
| Mean | 52.8 |  | 64.9 | 62.2 |  | 53.4 | 44.7 |  | 56.3 | 54.4 |  | 63.9 | 61.2 |  | 61.2 | 57.1 |  | 66.6 | 62.5 |
| SD | 15.6 |  | 11.4 | 14.2 |  | 16.6 | 15.0 |  | 12.0 | 12.6 |  | 11.9 | 13.7 |  | 11.2 | 62.5 |  | 15.5 | 16.7 |
| **SIMD 2016** |  |  |  |  |  |  |  |  |  |  |  |  |  |  |  |  |  |  |  |
| Mean | 4.8 |  | 5.8 | 5.2 |  | 5.6 | 4.6 |  | 5.7 | 4.9 |  | 5.7 | 4.9 |  | 5.6 | 4.9 |  | 5.5 | 4.7 |
| SD | 2.8 |  | 2.8 | 2.8 |  | 2.8 | 2.8 |  | 2.8 | 2.8 |  | 2.8 | 2.8 |  | 2.8 | 2.8 |  | 2.8 | 2.8 |
|  |  |  |  |  |  |  |  |  |  |  |  |  |  |  |  |  |  |  |  |
| **Matched comparators** |  |  |  |  |  |  |  |  |  |  |  |  |  |  |  |  |  |  |  |
| N with One | na |  | 25,555 | 2,392 |  | 73,996 | 6,329 |  | 130,110 | 6,818 |  | 57,986 | 3,468 |  | 211,282 | 16,387 |  | 39,801 | 6,225 |
| % with One | na |  | 100.0 | 28.2 |  | 100.0 | 7.0 |  | 100.0 | 14.1 |  | 100.0 | 14.1 |  | 100.0 | 25.0 |  | 100.0 | 18.2 |
| N with Two | na |  |  | 6,085 |  |  | 84,590 |  |  | 41,590 |  |  | 21,166 |  |  | 49,197 |  |  | 27,031 |
| % with Two | na |  |  | 71.8 |  |  | 93.0 |  |  | 85.9 |  |  | 85.9 |  |  | 75.0 |  |  | 81.8 |

“Other Antihypertensives” were defined on the basis of treatment with a combination of thiazide diuretics, diuretics, BBs, AA and/or CCBs, but not treatment with at least two of these groups within in the last 3 months of the eligible treatment window.

Table S6: Medical event and comorbidity variables, cohort 2

|  |  | Comparison Group | |  | Thiazide diuretics | |  | Beta Blockers | |  | Angiotensin Antagonists | |  | Calcium Channel Blockers | |  | Polytherapy | |  | Other Antihyper-tensives | |
| --- | --- | --- | --- | --- | --- | --- | --- | --- | --- | --- | --- | --- | --- | --- | --- | --- | --- | --- | --- | --- | --- |
|  |  | n | % |  | n | % |  | n | % |  | n | % |  | n | % |  | n | % |  | n | % |
| Total |  | 502,937 | 100 |  | 8,477 | 100 |  | 90,919 | 100 |  | 48,408 | 100 |  | 24,634 | 100 |  | 65,584 | 100 |  | 34,256 | 100 |
|  |  |  |  |  |  |  |  |  |  |  |  |  |  |  |  |  |  |  |  |  |  |
| **Survival events** |  |  |  |  |  |  |  |  |  |  |  |  |  |  |  |  |  |  |  |  |  |
| Treatment for depression |  | 196,587 | 39.1 |  | 6,127 | 72.3 |  | 72,024 | 79.2 |  | 32,794 | 67.8 |  | 16,054 | 65.2 |  | 41,685 | 63.6 |  | 22,289 | 65.1 |
| Treatment for Bipolar disorder |  | 39,998 | 8.0 |  | 900 | 10.6 |  | 10,543 | 11.6 |  | 4,018 | 8.3 |  | 2,445 | 9.9 |  | 4,817 | 7.3 |  | 5,321 | 15.5 |
| Died |  | 48,313 | 9.6 |  | 1,055 | 12.5 |  | 4,154 | 4.6 |  | 3,494 | 7.2 |  | 2,404 | 9.8 |  | 6,168 | 9.4 |  | 26,644 | 9.8 |
| Changed Therapy |  |  |  |  | 3,323 | 39.2 |  | 9,101 | 10.0 |  | 16,250 | 33.6 |  | 7,065 | 28.7 |  |  |  |  |  |  |
| **History of hospital treatment measure** |  |  |  |  |  |  |  |  |  |  |  |  |  |  |  |  |  |  |  |  |  |
| Cardiovascular disease |  | 18,452 | 3.7 |  | 801 | 9.5 |  | 7,245 | 8.0 |  | 6,368 | 13.2 |  | 3,380 | 13.7 |  | 22,541 | 34.4 |  | 6,435 | 18.8 |
| Substance Abuse |  | 84,585 | 16.8 |  | 1,068 | 12.6 |  | 16,402 | 18.0 |  | 9,116 | 18.8 |  | 4,694 | 19.1 |  | 18,479 | 28.2 |  | 9,264 | 27.0 |
| Head Injury |  | 29,036 | 5.8 |  | 303 | 3.6 |  | 5,552 | 6.1 |  | 2,517 | 5.2 |  | 1,167 | 4.7 |  | 3,403 | 5.2 |  | 1,870 | 5.5 |
| Self-harm |  | 37,894 | 7.5 |  | 389 | 4.6 |  | 10,182 | 11.2 |  | 3,016 | 6.2 |  | 1,491 | 6.1 |  | 3,692 | 5.6 |  | 2,738 | 8.0 |
| Schizophrenia |  | 14,363 | 2.9 |  | 176 | 2.1 |  | 1,981 | 2.2 |  | 1,172 | 2.4 |  | 582 | 2.4 |  | 1,069 | 1.6 |  | 1,035 | 3.0 |
| Major Depressive Disorder |  | 34,607 | 6.9 |  | 594 | 7.0 |  | 8,542 | 9.4 |  | 3,760 | 7.8 |  | 2,039 | 8.3 |  | 4,686 | 7.2 |  | 3,762 | 11.0 |
| Bipolar Disorder |  | 6,708 | 1.3 |  | 95 | 1.1 |  | 1,042 | 1.2 |  | 626 | 1.3 |  | 358 | 1.5 |  | 566 | 0.9 |  | 652 | 1.9 |
| Other mood disorder |  | 4,868 | 1.0 |  | 112 | 1.3 |  | 880 | 1.0 |  | 457 | 0.9 |  | 321 | 1.3 |  | 509 | 0.8 |  | 516 | 1.5 |
| Personality disorder |  | 7,019 | 1.4 |  | 94 | 1.1 |  | 1,847 | 2.0 |  | 620 | 1.3 |  | 342 | 1.4 |  | 761 | 1.2 |  | 744 | 2.2 |
| **History of Prescriptions** |  |  |  |  |  |  |  |  |  |  |  |  |  |  |  |  |  |  |  |  |  |
| Other Cardiovascular drugs |  | 71,980 | 14.3 |  | 3,455 | 40.8 |  | 19,286 | 21.2 |  | 24,738 | 51.1 |  | 12,206 | 49.6 |  | 42,175 | 64.3 |  | 15,581 | 45.5 |
| Diabetic drugs |  | 10,920 | 2.2 |  | 387 | 4.6 |  | 2,366 | 2.6 |  | 8,608 | 17.8 |  | 1,217 | 4.9 |  | 6,405 | 9.8 |  | 2,588 | 7.6 |
| Hypnotics and anxiolytics |  | 220,526 | 43.9 |  | 3,805 | 44.9 |  | 50,315 | 55.3 |  | 20,721 | 42.8 |  | 11,641 | 47.3 |  | 26,868 | 41.0 |  | 18,279 | 53.4 |
| Antidepressant Drugs |  | 347,152 | 69.0 |  | 6,370 | 75.1 |  | 76,640 | 84.3 |  | 35,738 | 73.8 |  | 18,444 | 74.9 |  | 42,813 | 65.3 |  | 24,591 | 71.8 |
| Psychoses and related disorders |  | 40,190 | 8.0 |  | 591 | 7.0 |  | 8,081 | 8.9 |  | 3,251 | 6.7 |  | 1,940 | 7.9 |  | 3,091 | 4.7 |  | 4,112 | 12.0 |
|  |  |  |  |  |  |  |  |  |  |  |  |  |  |  |  |  |  |  |  |  |  |

“Other Antihypertensives” were defined on the basis of treatment with a combination of thiazide diuretics, diuretics, BBs, AA and/or CCBs, but not treatment with at least two of these groups within in the last 3 months of the eligible treatment window.

Table S7: Number at risk at start of year, and number of first new onset of Major depressive disorder and Bipolar disorder

|  | Year 1 | Year 2 | Year 3 | Year 4 | Year 5 | Year 6 | Year 7 |
| --- | --- | --- | --- | --- | --- | --- | --- |
|  | N at Risk (N Failures) | N at Risk (N Failures) | N at Risk (N Failures) | N at Risk (N Failures) | N at Risk (N Failures) | N at Risk (N Failures) | N at Risk (N Failures) |
| **Cohort 1** | | | | | | | |
| **Major Depressive Disorder** | | | | | | | |
| Comparison | 538730 (23057) | 469614 (18799) | 405964 (15074) | 348452 (12655) | 295710 (11032) | 246646 (7756) | 202096 (5667) |
| Thiazide Diuretics | 25555 (1297) | 19930 (957) | 15961 (698) | 12725 (524) | 10143 (421) | 7854 (285) | 5797 (204) |
| Beta Blockers | 73996 (6619) | 55273 (3655) | 42852 (2464) | 33293 (1740) | 25418 (1285) | 18904 (755) | 13763 (477) |
| Angiotensin Antagonists | 130110 (5909) | 98380 (4156) | 77952 (2999) | 61780 (2313) | 48515 (1829) | 37245 (1214) | 27673 (802) |
| Calcium Channel Blockers | 57986 (2496) | 40413 (1716) | 29628 (1129) | 21775 (786) | 15811 (618) | 11172 (372) | 7822 (230) |
| Poltherapy | 211282 (10860) | 188908 (8799) | 168167 (7177) | 149290 (6138) | 131551 (5716) | 114360 (4566) | 98269 (3448) |
| Other Antihypertensives | 39801 (2855) | 31517 (2050) | 25094 (1438) | 19962 (1147) | 15645 (876) | 12004 (589) | 8829 (356) |
| **Bipolar Disorder** | | | | | | | |
| Comparison | 538730 (2251) | 489992 (2159) | 440705 (1949) | 392433 (1685) | 345094 (1554) | 298844 (1224) | 252665 (883) |
| Thiazide Diuretics | 2555 (74) | 21007 (84) | 17658 (73) | 14701 (76) | 12162 (75) | 9772 (44) | 7462 (46) |
| Beta Blockers | 73996 (213) | 61040 (212) | 50672 (157) | 41528 (159) | 33248 (131) | 25791 (102) | 19192 (59) |
| Angiotensin Antagonists | 130110 (198) | 103316 (248) | 85318 (219) | 70153 (180) | 57039 (165) | 45252 (149) | 34695 (95) |
| Calcium Channel Blockers | 57986 (166) | 42408 (157) | 32547 (158) | 24803 (119) | 18640 (94) | 13684 (79) | 9911 (55) |
| Poltherapy | 211282 (456) | 198948 (556) | 185439 (600) | 171613 (647) | 157346 (728) | 142594 (695) | 127244 (630) |
| Other Antihypertensives | 39801 (432) | 33924 (375) | 28839 (307) | 24304 (217) | 20179 (175) | 16380 (142) | 12710 (79) |
| Cohort 2 | | | | | | | |
| **Major Depressive Disorder** | | | | | | | |
| Comparison | 502937 (166159) | 275860 (11728) | 218451 (7164) | 170967 (4726) | 129132 (3440) | 91605 (2078) | 58853 (1169) |
| Thiazide Diuretics | 8477 (4966) | 2597 (296) | 1805 (176) | 1253 (110) | 836 (75) | 545 (33) | 322 (18) |
| Beta Blockers | 90919 (63722) | 20930 (3537) | 13601 (1739) | 9026 (951) | 5969 (585) | 3719 (292) | 2150 (123) |
| Angiotensin Antagonists | 48408 (26766) | 15301 (1802) | 10270 (894) | 6975 (548) | 4628 (343) | 2960 (167) | 1693 (77) |
| Calcium Channel Blockers | 24634 (13459) | 7286 (806) | 4621 (413) | 2865 (223) | 1668 (137) | 935 (55) | 498 (25) |
| Poltherapy | 65584 (33393) | 28086 (3198) | 21291 (1967) | 16279 (1358) | 11977 (920) | 8413 (521) | 5561 (322) |
| Other Antihypertensives | 34256 (19109) | 11063 (1417) | 7350 (789) | 4912 (458) | 3204 (276) | 1966 (144) | 1094 (68) |
| **Bipolar Disorder** | | | | | | | |
| Comparison | 502937 (31457) | 388591 (3252) | 317545 (1934) | 254160 (1365) | 195952 (948) | 142943 (563) | 95775 (330) |
| Thiazide Diuretics | 9477 (541) | 6198 (76) | 4905 (59) | 3881 (47) | 2952 (42) | 2147 (25) | 1393 (6) |
| Beta Blockers | 90919 (7064) | 68267 (1156) | 53520 (796) | 41064 (506) | 30536 (374) | 21133 (208) | 13526 (128) |
| Angiotensin Antagonists | 48408 (2794) | 33754 (287) | 25648 (207) | 19083 (154) | 13817 (91) | 9561 (64) | 6150 (27) |
| Calcium Channel Blockers | 24634 (1686) | 15998 (199) | 11484 (135) | 7937 (78) | 5267 (51) | 3372 (39) | 2010 (20) |
| Poltherapy | 65584 (2889) | 55916 (539) | 48307 (385) | 41137 (333) | 34066 (288) | 27117 (228) | 20662 (145) |
| Other Antihypertensives | 34256 (3822) | 24054 (557 | 18742 (360) | 14474 (239) | 10787 (176) | 7660 (91) | 4904 (41) |

“Other Antihypertensives” were defined on the basis of treatment with a combination of thiazide diuretics, diuretics, BBs, AA and/or CCBs, but not treatment with at least two of these groups within in the last 3 months of the eligible treatment window.

Figure S1. Flow chart for derivation of cohort 1 sample

Cohort or control had a death record

N = 642

Eligible only before 01/01/2010

N = 196,085

No death record prior to eligibility

N = 548,777

Men 289,862, Women 258,915

Age < 18: N = 6,261

Age > 10: N = 203

Age missing: N = 205

Matched to controls

N = 549,419,

Men 290,216, Women 259,203

Meet original eligibility criteria

N= 968,930

Men 490,190, Women 478,740

Eligible period after 31/12/2009

N= 772,845

Men 396,604, Women 376,241

Excluded as no follow up data.

N= 3,322

Not matched

N = 223,426

Aged between 18 and 100

N = 542,108

Men 287,095, Women 255,013

Final Sample

N= 538,796

Thiazide diuretics 25,555; Beta blockers 74,028; Angiotensin antagonists 130,123

Calcium Channel Blockers 57,992; Polytherapy 211,293; Other Antihypertensives 39,805.

Had eligible date before 31/12/2016

N = 538,796

Men 285,361, Women 253,435

Figure S2: Flow chart for derivation of cohort 2 sample

Cohort or control death record prior to eligibility

N = 1,183

Eligible only before 01/01/2010

N = 71,968

No Death record prior to eligibility

N =276,404:

Men 109,510, Women 166,894

Excluded < 18 or >100 Yrs

N =1,167

Matched to controls

N = 277,587

Men 110,091, Women 167,496

Meet Original Eligibility Criteria

N = 555,975

Men 217,000, Women 338,975

Eligible period after 31/12/2009

N= 484,007

Men 188,936, Women 295,071

Excluded as no follow up data.

N= 3,322

Not matched

N = 206,420

Aged between 18 and 100

N= 275,160

Men 109,160, Women166,077

Final Sample:

N= 272,278

Thiazide diuretics 8,477; Beta blockers 90,919; Angiotensin antagonists 48,408;

Calcium Channel Blockers 24,634; Polytherapy 65,584; Other antihyperetensives 34,256

Two controls 230,659, One control 41,619

Had eligible date before 31/12/2016

N = 272,278

Men 108,005, Women 164,273

Figure S3: First onset of mood disorders, as indicated by receipt of prescriptions or admission to hospital, by therapy class (people with mental illness).

*
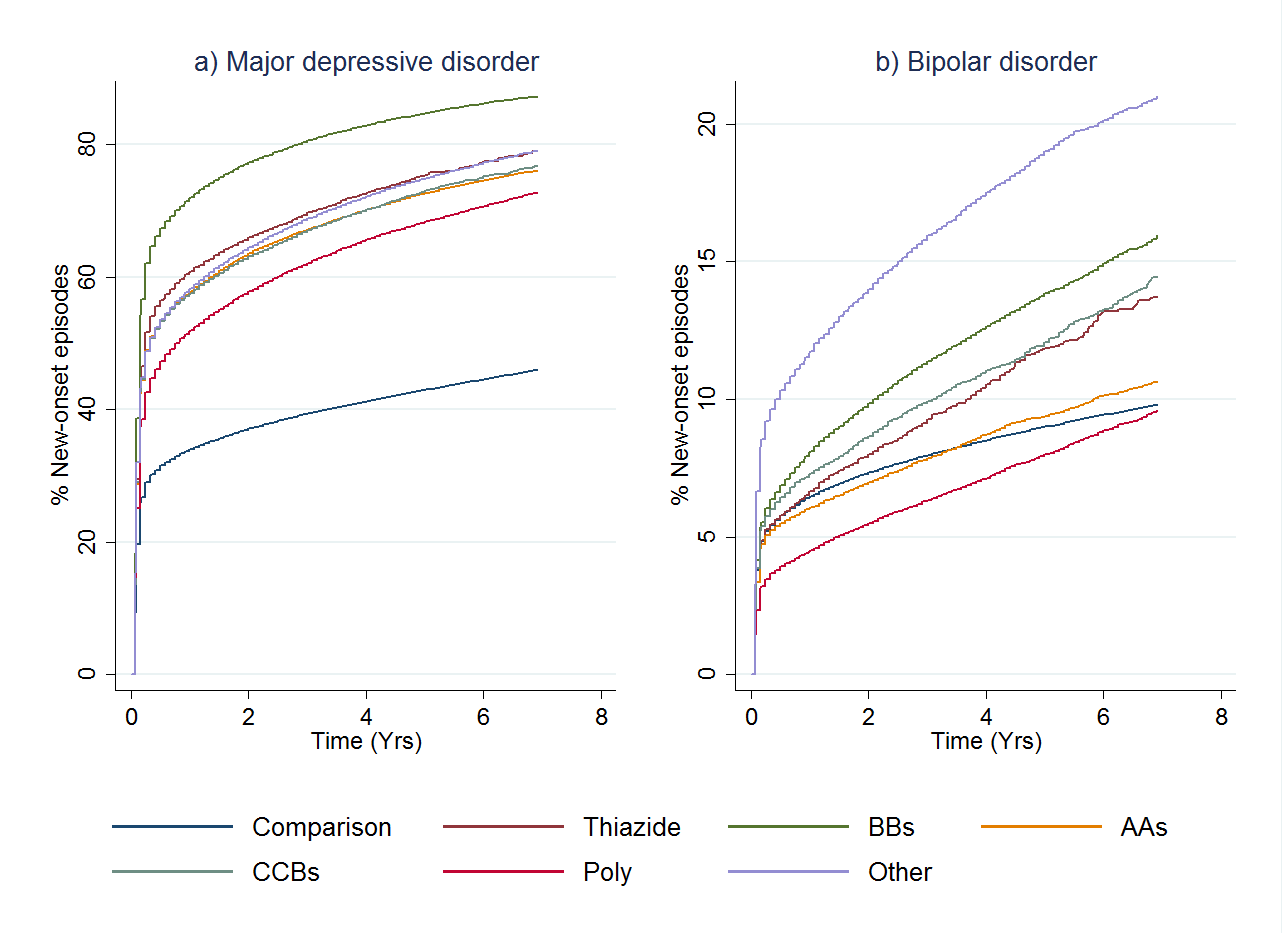
*

AA – Angiotensin Antagonists, BB – Beta Blockers, CCB – Calcium Channel Blockers

Figure S3 shows cumulative distributions functions for major depressive disorder (panel a) and bipolar disorder (panel b) by therapy class for people with a history of treatment for mental illness (cohort 2). “Other Antihypertensives” were defined on the basis of treatment with a combination of thiazide diuretics, diuretics, BBs, AA and/or CCBs, but not treatment with at least two of these groups within in the last 3 months of the eligible treatment window.

Figure S4: Hazard ratios for new onset depression, as indicated by receipt of prescriptions or admission to hospital, by therapy class (men without mental illness).


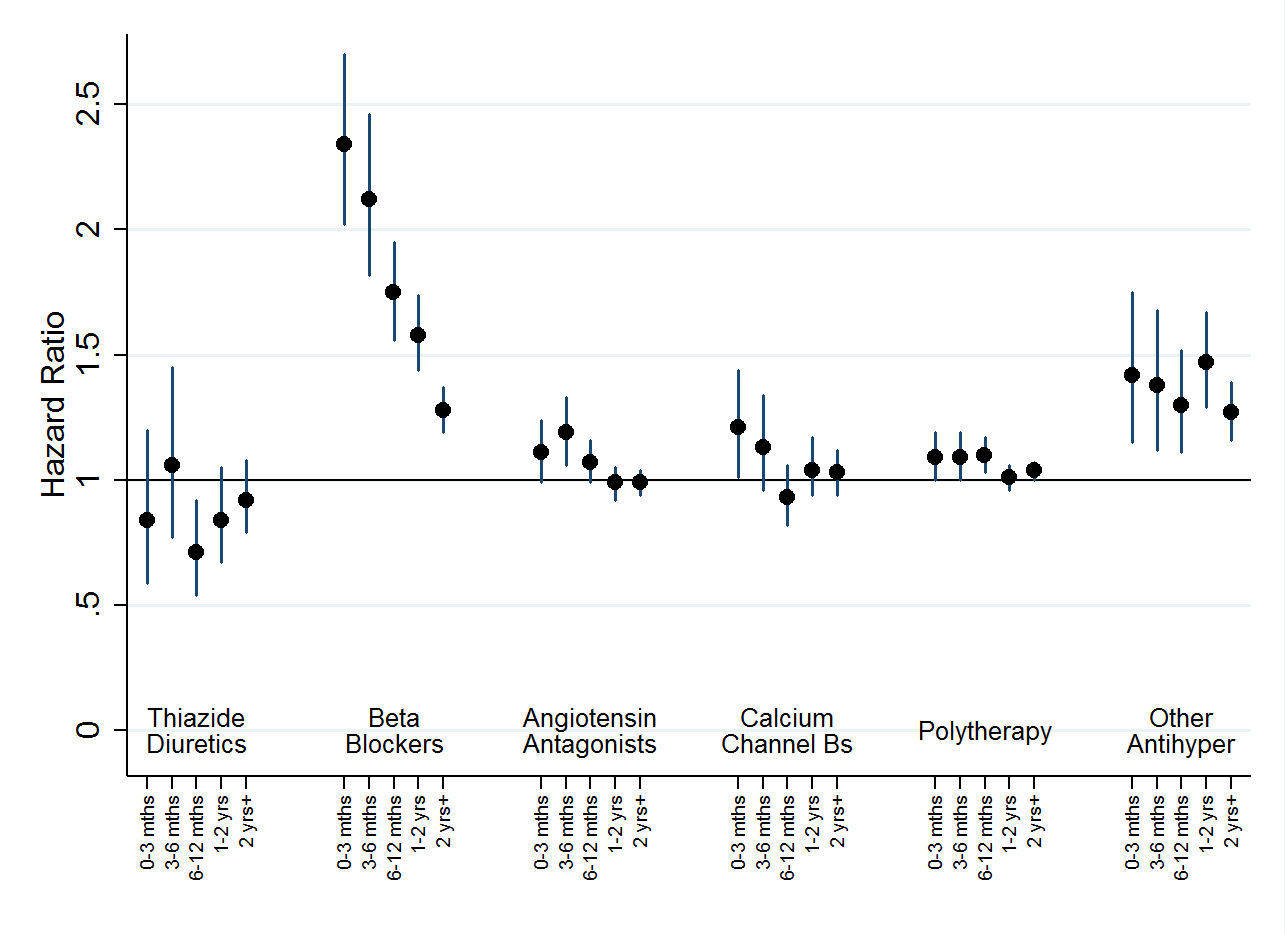


Figure S4 shows hazard ratios for new onset depression by therapy class for men without a history of treatment for mental illness (Cohort 1). Adjustment was carried out for hospital treatment for cardiovascular disease, substance abuse, head injury, self-harm, and pharmaceutical treatment for other cardiovascular drugs and diabetic drugs. “Other Antihypertensives” were defined on the basis of treatment with a combination of thiazide diuretics, diuretics, BBs, AA and/or CCBs, but not treatment with at least two of these groups within in the last 3 months of the eligible treatment window.

Figure S5: Hazard ratios for new onset depression, as indicated by receipt of prescriptions or admission to hospital, by therapy class (women without mental illness).


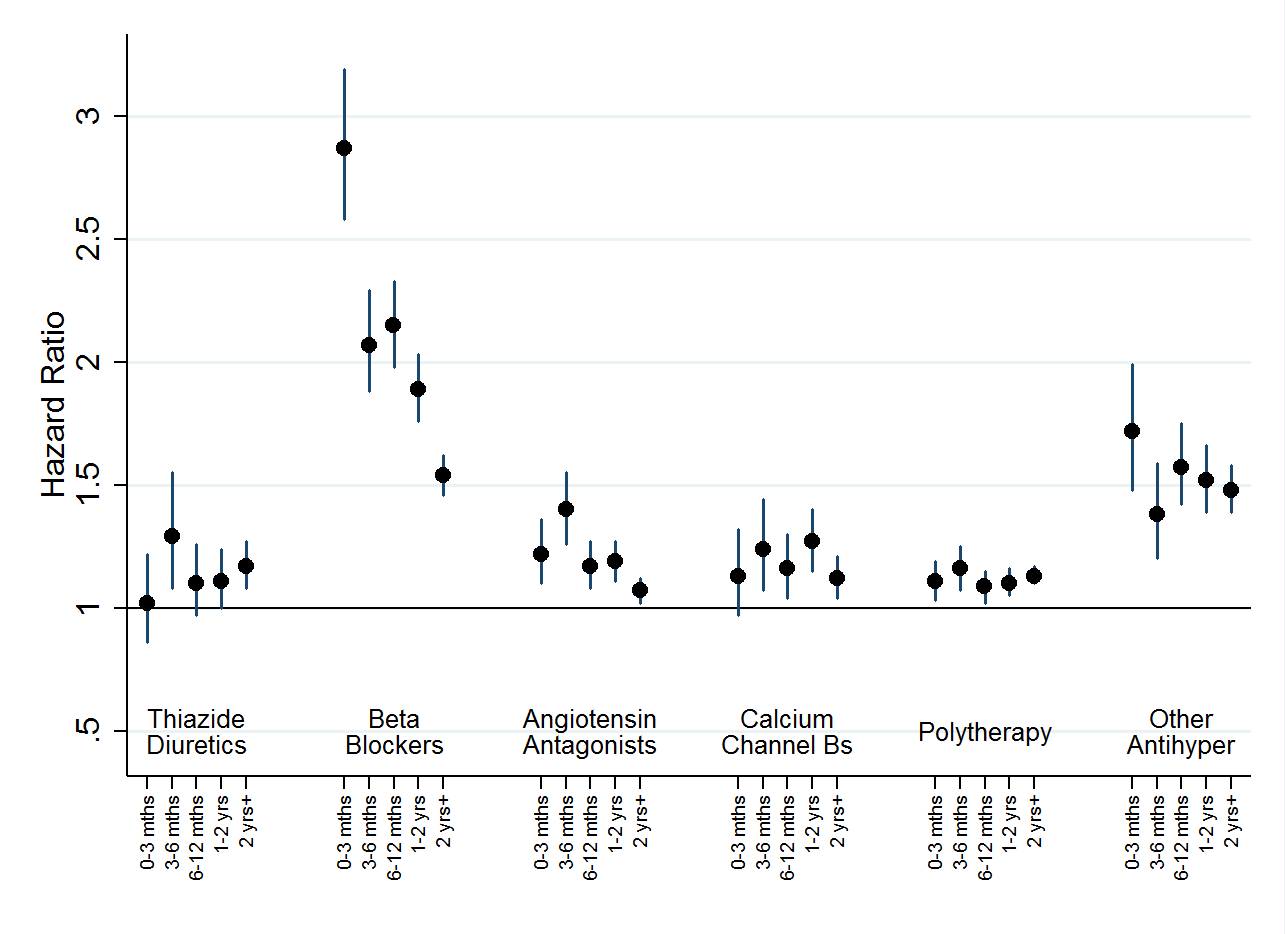
 Figure S5 shows hazard ratios for new onset depression by therapy class for women without a history of treatment for mental illness (Cohort 1). Adjustment was carried out for hospital treatment for cardiovascular disease, substance abuse, head injury, self-harm, and pharmaceutical treatment for other cardiovascular drugs and diabetic drugs. “Other Antihypertensives” were defined on the basis of treatment with a combination of thiazide diuretics, diuretics, BBs, AA and/or CCBs, but not treatment with at least two of these groups within in the last 3 months of the eligible treatment window.

Figure S6: Hazard ratios for new onset depression, as indicated by receipt of prescriptions or admission to hospital, by therapy class (people with mental illness).


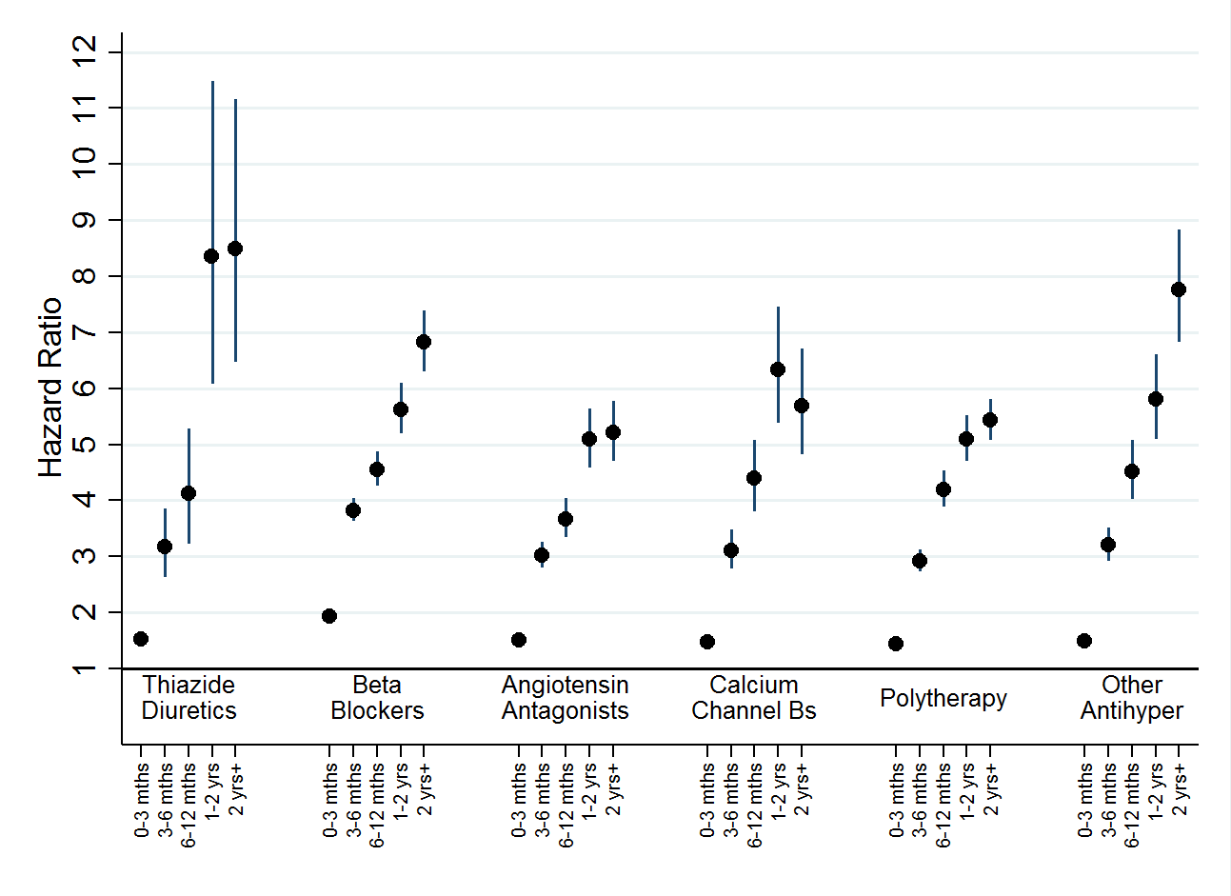


Figure S6: shows hazard ratios for new onset depression by therapy class for people without a history of treatment for mental illness (Cohort 2). Adjustment was carried out for hospital treatment for cardiovascular disease, substance abuse, head injury, self-harm, schizophrenia, major depressive disorder, bipolar disorder, other mood disorders and personality disorder, and pharmaceutical treatment for other cardiovascular drugs, diabetic drugs, hypnotics and anxiolytics, antidepressant drugs, psychoses and related disorders. “Other Antihypertensives” were defined on the basis of treatment with a combination of thiazide diuretics, diuretics, BBs, AA and/or CCBs, but not treatment with at least two of these groups within in the last 3 months of the eligible treatment window.

Figure S7: Hazard ratios for new onset bipolar disorder, as indicated by receipt of prescriptions or admission to hospital, by therapy class (men without mental illness).


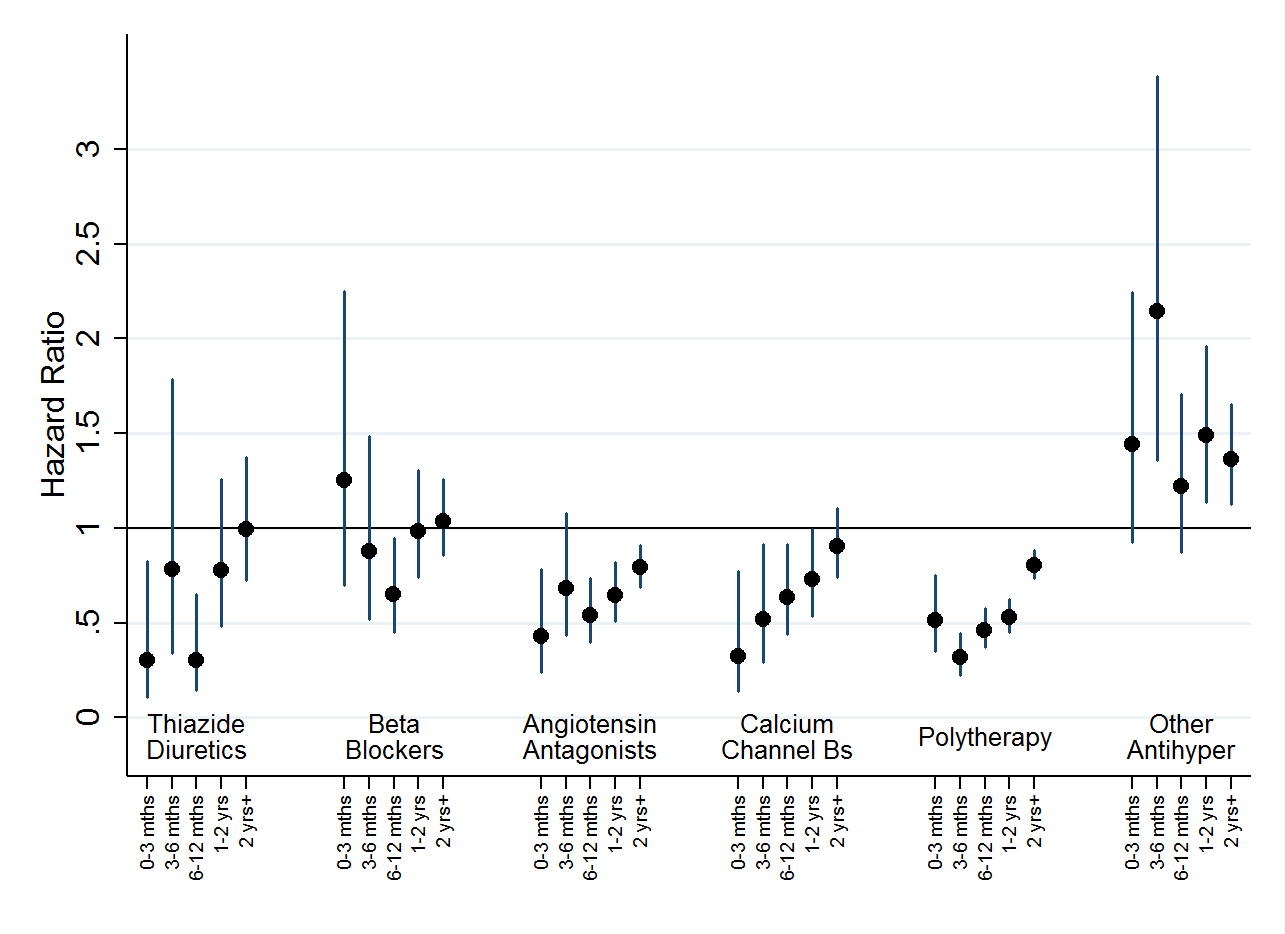


Figure S7 shows hazard ratios for new onset depression by therapy class for men without a history of treatment for mental illness (Cohort 1). Adjustment was carried out for hospital treatment for cardiovascular disease, substance abuse, head injury, self-harm, and pharmaceutical treatment for other cardiovascular drugs and diabetic drugs. “Other Antihypertensives” were defined on the basis of treatment with a combination of thiazide diuretics, diuretics, BBs, AA and/or CCBs, but not treatment with at least two of these groups within in the last 3 months of the eligible treatment window.

Figure S8: Hazard ratios for new onset bipolar disorder, as indicated by receipt of prescriptions or admission to hospital, by therapy class (women without mental illness).


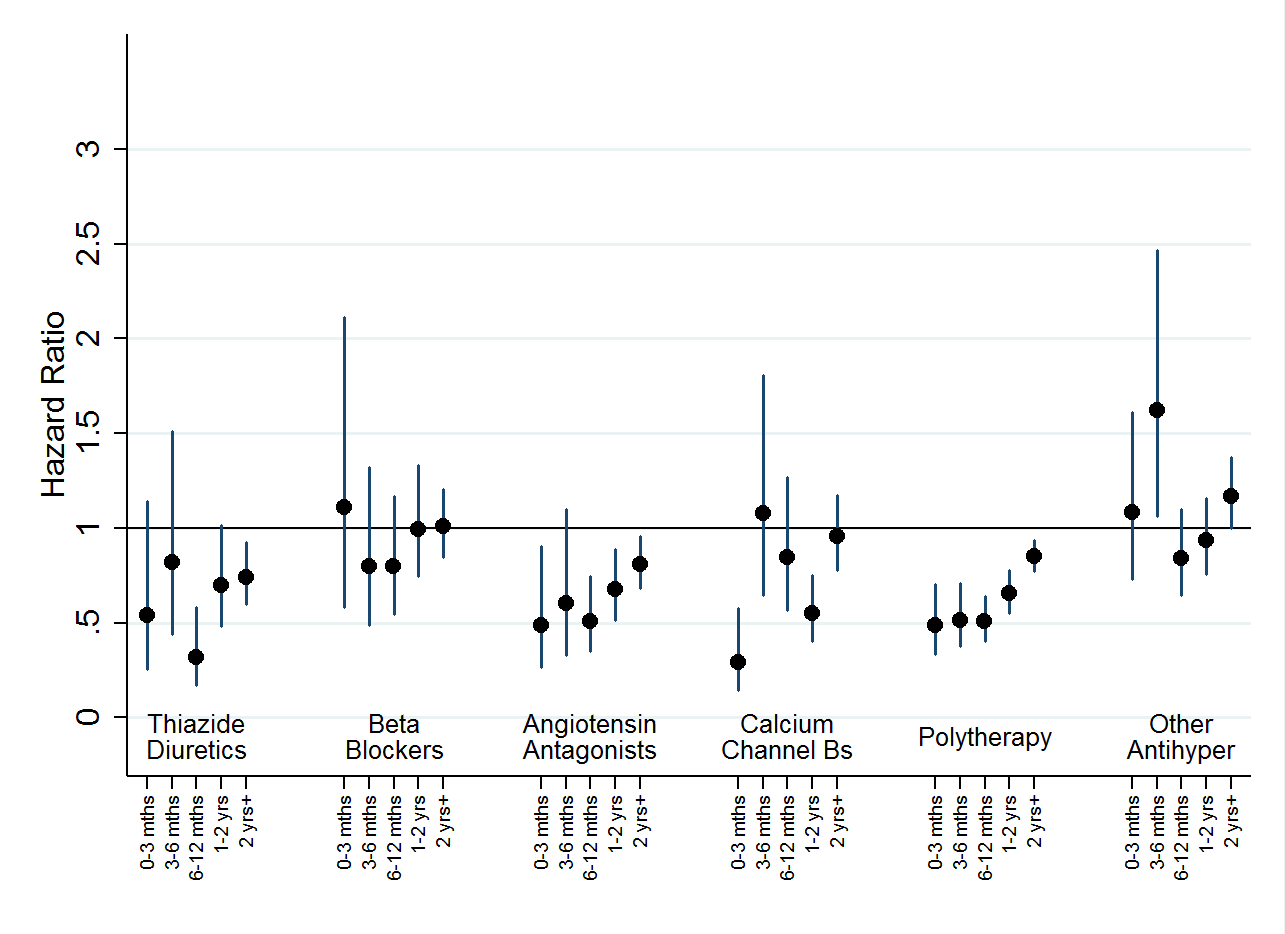


Figure S8 shows hazard ratios for new onset depression by therapy class for women without a history of treatment for mental illness (Cohort 1). Adjustment was carried out for hospital treatment for cardiovascular disease, substance abuse, head injury, self-harm, and pharmaceutical treatment for other cardiovascular drugs and diabetic drugs. “Other Antihypertensives” were defined on the basis of treatment with a combination of thiazide diuretics, diuretics, BBs, AA and/or CCBs, but not treatment with at least two of these groups within in the last 3 months of the eligible treatment window.

Figure S9: Hazard ratios for new onset bipolar disorder, as indicated by receipt of prescriptions or admission to hospital, by therapy class (people with mental illness).


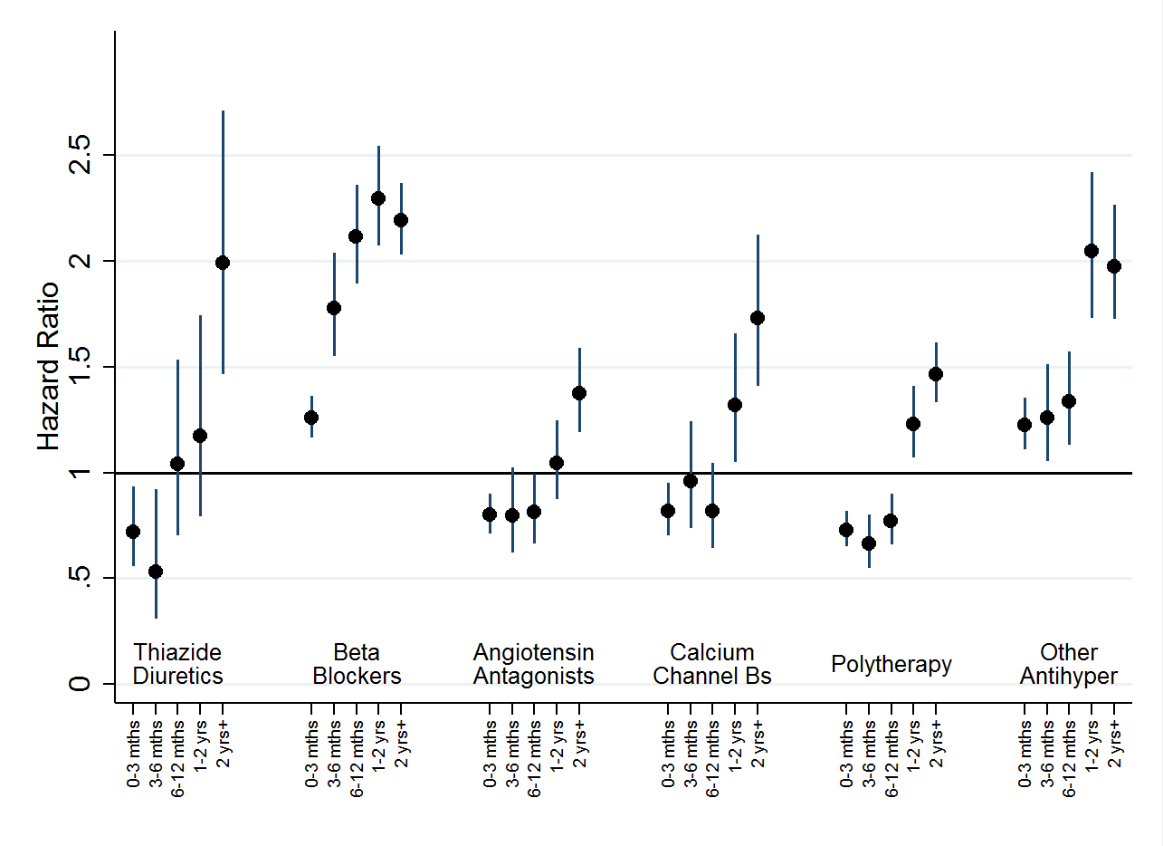


Figure S9 shows hazard ratios for new onset depression by therapy class for people without a history of treatment for mental illness (Cohort 2). Adjustment was carried out for hospital treatment for cardiovascular disease, substance abuse, head injury, self-harm, schizophrenia, major depressive disorder, bipolar disorder, other mood disorders and personality disorder, and pharmaceutical treatment for other cardiovascular drugs, diabetic drugs, hypnotics and anxiolytics, antidepressant drugs, psychoses and related disorders. “Other Antihypertensives” were defined on the basis of treatment with a combination of thiazide diuretics, diuretics, BBs, AA and/or CCBs, but not treatment with at least two of these groups within in the last 3 months of the eligible treatment window.
